# 1,3-Dibenzyl-1,2,3,4-tetra­hydro­quinazoline-2,4-dione

**DOI:** 10.1107/S1600536810019938

**Published:** 2010-06-05

**Authors:** Gavhar Karimova, Jamshid Ashurov, Nasir Mukhamedov, Nusrat A. Parpiev, Khusniddin M. Shakhidoyatov

**Affiliations:** a The Mirzo Ulugbek National University of Uzbekistan, Faculty of Chemistry, University Str. 6, Tashkent 100779, Uzbekistan; b Institute of Bioorganic Chemistry, Academy of Sciences of Uzbekistan, Mirzo Ulugbek Str. 83, Tashkent, 100125 Uzbekistan; c S. Yunusov Institute of the Chemistry of Plant Substances, Academy of Sciences of Uzbekistan, Mirzo Ulugbek Str. 77, Tashkent 100170, Uzbekistan

## Abstract

The asymmetric unit of the title compound, C_22_H_18_N_2_O_2_, contains two independent mol­ecules, which differ in the orientations of the benzyl groups with respect to the planar (r.m.s. deviations of 0.031 and 0.020 Å) quinazoline-2,4-dione skeletons [dihedral angles of 73.97 (4) and 70.07 (4)° in the first mol­ecule and 75.63 (4) and 63.52 (3)° in the second]. The crystal structure is stabilized by weak inter­molecular C—H⋯O and C—H⋯π interactions and aromatic π–π stacking inter­actions [centroid–centroid distance = 3.735 (2) Å].

## Related literature

For the synthesis of the title compound, see: Hedayatullah (1981 ▶). For the synthesis of quinazoline-2,4-dione derivatives, see: Shi *et al.* (2007 ▶); Kuryazov *et al.* (2008 ▶). For the biological activity of quinazoline-2,4-dione derivatives, see: Colottaa *et al.* (2004 ▶); Yakhontov *et al.* (1977 ▶). For related structures, see: Mazza *et al.* (1988 ▶). For bond-length data, see: Allen *et al.* (1987 ▶).
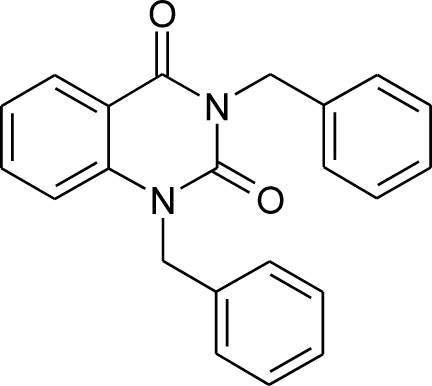


## Experimental

### 

#### Crystal data

C_22_H_18_N_2_O_2_
*M* *_r_* = 342.38
Orthorhombic, 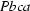

*a* = 17.8989 (4) Å
*b* = 14.0071 (4) Å
*c* = 27.7222 (6) Å
*V* = 6950.3 (3) Å^3^
*Z* = 16
Cu *K*α radiation
μ = 0.68 mm^−1^
*T* = 293 K
0.5 × 0.4 × 0.35 mm

#### Data collection

Oxford Diffraction Xcalibur Ruby diffractometer
Absorption correction: multi-scan (*CrysAlis PRO*; Oxford Diffraction, 2009 ▶) *T*_min_ = 0.804, *T*_max_ = 1.000
18947 measured reflections
7088 independent reflections
4141 reflections with *I* > 2σ(*I*)
*R*_int_ = 0.030

#### Refinement

*R*[*F* ^2^ > 2σ(*F* ^2^)] = 0.041
*wR*(*F* ^2^) = 0.112
*S* = 0.90
7088 reflections
470 parameters
H-atom parameters constrained
Δρ_max_ = 0.28 e Å^−3^
Δρ_min_ = −0.14 e Å^−3^

### 

Data collection: *CrysAlis PRO* (Oxford Diffraction, 2009 ▶); cell refinement: *CrysAlis PRO*; data reduction: *CrysAlis PRO*; program(s) used to solve structure: *SHELXS97* (Sheldrick, 2008 ▶); program(s) used to refine structure: *SHELXL97* (Sheldrick, 2008 ▶); molecular graphics: *XP* in *SHELXTL* (Sheldrick, 2008 ▶); software used to prepare material for publication: *SHELXL97*.

## Supplementary Material

Crystal structure: contains datablocks I, global. DOI: 10.1107/S1600536810019938/fj2301sup1.cif

Structure factors: contains datablocks I. DOI: 10.1107/S1600536810019938/fj2301Isup2.hkl

Additional supplementary materials:  crystallographic information; 3D view; checkCIF report

## Figures and Tables

**Table 1:** Hydrogen-bond geometry (Å, °) *Cg*4 and *Cg*8 are the centroids of the C17*A*–C22*A* and C10*B*–C15*B* rings, respectively.

*D*—H⋯*A*	*D*—H	H⋯*A*	*D*⋯*A*	*D*—H⋯*A*
C19*A*—H19*A*⋯O2b^i^	0.93	2.70	3.419 (3)	134
C6*B*—H6*B*⋯C11a^ii^	0.93	2.89	3.604 (3)	134
C21*B*—H21*B*⋯C11a^iii^	0.93	2.80	3.600 (3)	145
C19*A*—H19*A*⋯O2b^i^	0.93	2.70	3.419 (3)	134
C7*B*—H7*B*⋯*Cg*4	0.93	2.78	3.586 (2)	146
C5*A*—H5*A*⋯*Cg*8^ii^	0.93	2.90	3.641 (2)	138

Symmetry codes: (i) 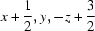; (ii) 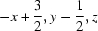; (iii) 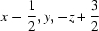.

**Fig. 1.**
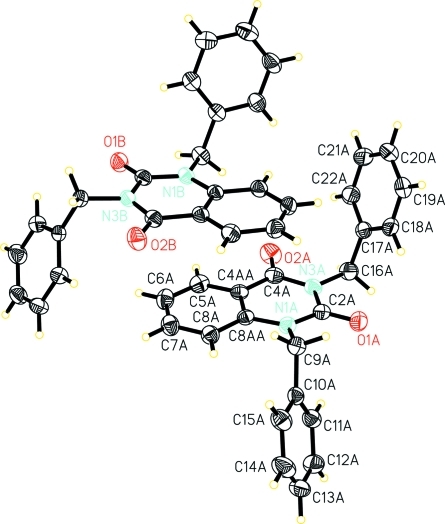
Asymmetric unit of cell with atom labels and 30% probability displacement ellipsoids for non-H atoms.

## supplementary crystallographic information

### Comment

Quinazoline-2,4-diones have been frequently used as intermediates and synthetic precursors for the preparation of a wide variety of heterocyclic compounds (Kuryazov *et al.*, 2008). In addition, they possess different biological activities (Colottaa *et al.*, 2004; Yakhontov *et al.*, 1977).

The title compound consists of a quinazoline-2,4-dione skeleton with two benzyl groups. The asymmetric unit contains two molecules of 1,2,3,4-tetrahydro-1,3-dibenzylquinazoline-2,4-dione (Fig.1). Orientation of benzyl groups with respect to the planar quinazoline-2,4-dione skeletons are different for independent molecules. Dihedral angles between planar quinazoline-2,4-dione system and benzyl group planes are 73.97 (4)° and 70.07 (4)° (for molecule A) and 75.63 (4)° and 63.52 (3)° (for molecule B). Torsion angles responsible for orientation of benzyl groups are shown in table 1. (In order compare torsions between A and B independent molecules must be taken absolute values of torsion angles).

Quinazoline-2,4-dione system of the molecules are packed into sheets along *b* axis by a aromatic π–π stacking interaction. The benzene rings in the quinazoline-2,4-dione system standing nearly parallel (molecules A and B) are separated with distance of 3.477 (2) Å and benzene ring-centroid separation is 3.735 (2) Å with ring offset of 1.364 (2) Å. This distances for molecules of A at (*x*,*y*,*z*) and B at (1.5 - *x*, 1/2 + *y*, *z*) are 3.493 (2) Å, 3.791 (2) Å and 1.473 (2) Å.

The observed structure is stabilized by weak C—H···O and C—H···C_ar_ type hydrogen bonds (Table 2). The bond distances and angles in organic compound molecules are in normal ranges (Allen *et al.*, 1987).

### Experimental

To suspension of 1*H*-quinazoline-2,4-dione (1.62 g) in 40 ml benzene was added 10% aqueous solution of sodium hydroxide (40 ml), tetrabutylammonium bromide (1.29 g, 4 mmol) and benzyl chloride (3.80 g, 30 mmol). The mixture was heated until 60° C and helded out for 6 h (Hedayatullah, 1981). The organic layer was separated, washed with water until neutral reaction and dryed with Na_2_SO_4_, benzene was evaporated. Residue was recrystallized from benzene and obtained in 88% yield (3.02 g) of title compound. Colorless crystals suitable for X-ray analysis were obtained from dimethylformamide by slow evaporation.

### Refinement

Carbon-bound H atoms were positioned geometrically and treated as riding on their C atoms, with C—H distances of 0.93 Å (aromatic) and 0.97 Å (CH_2_) and were refined with *U*_iso_(H)=1.2Ueq(C).

### Crystal data

**Table:**

C_22_H_18_N_2_O_2_	*D*_x_ = 1.309 Mg m^−^^3^
*M**_r_* = 342.38	Melting point: 398(2) K
Orthorhombic, *P**b**c**a*	Cu *K*α radiation, λ = 1.54184 Å
Hall symbol: -P 2ac 2ab	Cell parameters from 2463 reflections
*a* = 17.8989 (4) Å	θ = 3.5–35.8°
*b* = 14.0071 (4) Å	µ = 0.68 mm^−^^1^
*c* = 27.7222 (6) Å	*T* = 293 K
*V* = 6950.3 (3) Å^3^	Prism, colourless
*Z* = 16	0.5 × 0.4 × 0.35 mm
*F*(000) = 2880	

### Data collection

**Table:**

Oxford Diffraction Xcalibur Ruby diffractometer	7088 independent reflections
Radiation source: Enhance (Cu) X-ray Source	4141 reflections with *I* > 2σ(*I*)
graphite	*R*_int_ = 0.030
Detector resolution: 10.2576 pixels mm^-1^	θ_max_ = 75.8°, θ_min_ = 4.0°
ω scans	*h* = −22→18
Absorption correction: multi-scan (*CrysAlis PRO*; Oxford Diffraction, 2009)	*k* = −11→17
*T*_min_ = 0.804, *T*_max_ = 1.000	*l* = −31→34
18947 measured reflections	

### Refinement

**Table:**

Refinement on *F*^2^	Secondary atom site location: difference Fourier map
Least-squares matrix: full	Hydrogen site location: inferred from neighbouring sites
*R*[*F*^2^ > 2σ(*F*^2^)] = 0.041	H-atom parameters constrained
*wR*(*F*^2^) = 0.112	*w* = 1/[σ^2^(*F*_o_^2^) + (0.0587*P*)^2^] where *P* = (*F*_o_^2^ + 2*F*_c_^2^)/3
*S* = 0.90	(Δ/σ)_max_ = 0.002
7088 reflections	Δρ_max_ = 0.28 e Å^−^^3^
470 parameters	Δρ_min_ = −0.14 e Å^−^^3^
0 restraints	Extinction correction: *SHELXL97* (Sheldrick, 2008), Fc^*^=kFc[1+0.001xFc^2^λ^3^/sin(2θ)]^-1/4^
Primary atom site location: structure-invariant direct methods	Extinction coefficient: 0.00081 (5)

### Special details

**Table:**

Geometry. All e.s.d.'s (except the e.s.d. in the dihedral angle between two l.s. planes) are estimated using the full covariance matrix. The cell e.s.d.'s are taken into account individually in the estimation of e.s.d.'s in distances, angles and torsion angles; correlations between e.s.d.'s in cell parameters are only used when they are defined by crystal symmetry. An approximate (isotropic) treatment of cell e.s.d.'s is used for estimating e.s.d.'s involving l.s. planes.
Refinement. Refinement of *F*^2^ against ALL reflections. The weighted *R*-factor *wR* and goodness of fit *S* are based on *F*^2^, conventional *R*-factors *R* are based on *F*, with *F* set to zero for negative *F*^2^. The threshold expression of *F*^2^ > σ(*F*^2^) is used only for calculating *R*-factors(gt) *etc*. and is not relevant to the choice of reflections for refinement. *R*-factors based on *F*^2^ are statistically about twice as large as those based on *F*, and *R*- factors based on ALL data will be even larger.

### Fractional atomic coordinates and isotropic or equivalent isotropic displacement parameters (Å^2^)

**Table:**

	*x*	*y*	*z*	*U*_iso_*/*U*_eq_	
O1A	0.90039 (7)	0.27786 (11)	0.72998 (5)	0.0745 (4)	
O2A	0.85328 (8)	0.30319 (11)	0.89063 (5)	0.0770 (4)	
N1A	0.77945 (8)	0.27259 (10)	0.75417 (5)	0.0498 (4)	
N3A	0.87693 (8)	0.29234 (10)	0.81052 (6)	0.0531 (4)	
C2A	0.85450 (10)	0.28112 (13)	0.76252 (7)	0.0538 (5)	
C4A	0.82872 (11)	0.29654 (13)	0.84973 (7)	0.0542 (5)	
C4AA	0.74920 (10)	0.29394 (11)	0.83793 (6)	0.0476 (4)	
C5A	0.69582 (11)	0.30185 (13)	0.87449 (7)	0.0596 (5)	
H5A	0.7113	0.3091	0.9063	0.072*	
C6A	0.62102 (11)	0.29916 (14)	0.86422 (8)	0.0639 (5)	
H6A	0.5859	0.3033	0.8888	0.077*	
C7A	0.59835 (11)	0.29017 (13)	0.81658 (8)	0.0627 (5)	
H7A	0.5476	0.2894	0.8094	0.075*	
C8A	0.64939 (10)	0.28232 (13)	0.77977 (7)	0.0563 (5)	
H8A	0.6332	0.2767	0.7480	0.068*	
C8AA	0.72575 (9)	0.28287 (11)	0.79034 (6)	0.0454 (4)	
C9A	0.75706 (10)	0.24198 (13)	0.70537 (6)	0.0531 (4)	
H9AA	0.7164	0.1965	0.7084	0.064*	
H9AB	0.7988	0.2089	0.6906	0.064*	
C10A	0.73253 (9)	0.32101 (13)	0.67192 (6)	0.0480 (4)	
C11A	0.76693 (11)	0.40913 (13)	0.67155 (7)	0.0583 (5)	
H11A	0.8039	0.4228	0.6941	0.070*	
C12A	0.74673 (12)	0.47754 (15)	0.63777 (8)	0.0722 (6)	
H12A	0.7701	0.5368	0.6377	0.087*	
C13A	0.69229 (13)	0.45750 (18)	0.60452 (8)	0.0775 (7)	
H13A	0.6785	0.5035	0.5820	0.093*	
C14A	0.65844 (13)	0.37067 (19)	0.60429 (8)	0.0801 (7)	
H14A	0.6220	0.3572	0.5814	0.096*	
C15A	0.67784 (11)	0.30240 (16)	0.63795 (7)	0.0671 (5)	
H15A	0.6539	0.2434	0.6378	0.081*	
C16A	0.95826 (10)	0.29432 (13)	0.81973 (8)	0.0627 (5)	
H16A	0.9681	0.3332	0.8480	0.075*	
H16C	0.9834	0.3235	0.7925	0.075*	
C17A	0.98951 (9)	0.19523 (13)	0.82771 (7)	0.0530 (4)	
C18A	1.00645 (10)	0.13649 (14)	0.78927 (8)	0.0617 (5)	
H18A	1.0011	0.1593	0.7579	0.074*	
C19A	1.03111 (11)	0.04455 (16)	0.79674 (9)	0.0748 (6)	
H19A	1.0409	0.0049	0.7706	0.090*	
C20A	1.04120 (12)	0.01171 (18)	0.84297 (10)	0.0873 (7)	
H20A	1.0579	−0.0503	0.8481	0.105*	
C21A	1.02667 (12)	0.07020 (18)	0.88159 (9)	0.0853 (7)	
H21A	1.0346	0.0482	0.9128	0.102*	
C22A	1.00042 (10)	0.16140 (16)	0.87410 (7)	0.0684 (6)	
H22A	0.9900	0.2005	0.9004	0.082*	
O1B	0.61180 (7)	0.06448 (9)	0.98324 (5)	0.0633 (4)	
O2B	0.54505 (7)	0.03102 (10)	0.82607 (5)	0.0668 (4)	
N1B	0.70512 (7)	0.06323 (10)	0.92718 (5)	0.0467 (3)	
N3B	0.57889 (7)	0.04627 (9)	0.90467 (5)	0.0455 (3)	
C2B	0.63124 (9)	0.05873 (12)	0.94108 (7)	0.0480 (4)	
C4AB	0.67396 (9)	0.04264 (11)	0.84349 (6)	0.0444 (4)	
C4B	0.59496 (9)	0.03999 (12)	0.85581 (6)	0.0476 (4)	
C5B	0.69580 (10)	0.03363 (12)	0.79517 (6)	0.0542 (5)	
H5B	0.6597	0.0278	0.7712	0.065*	
C6B	0.76984 (11)	0.03330 (13)	0.78293 (7)	0.0599 (5)	
H6B	0.7842	0.0264	0.7509	0.072*	
C7B	0.82289 (10)	0.04332 (13)	0.81853 (7)	0.0576 (5)	
H7B	0.8732	0.0429	0.8102	0.069*	
C8AB	0.72776 (9)	0.05321 (11)	0.87922 (6)	0.0435 (4)	
C8B	0.80291 (9)	0.05388 (12)	0.86611 (7)	0.0533 (4)	
H8B	0.8396	0.0615	0.8896	0.064*	
C9B	0.76085 (10)	0.07851 (12)	0.96557 (6)	0.0545 (5)	
H9B	0.7359	0.1041	0.9938	0.065*	
H9D	0.7968	0.1257	0.9547	0.065*	
C10B	0.80190 (10)	−0.01121 (13)	0.97963 (6)	0.0498 (4)	
C11B	0.76367 (11)	−0.08904 (13)	0.99740 (7)	0.0588 (5)	
H11B	0.7120	−0.0862	1.0005	0.071*	
C12B	0.80122 (13)	−0.17137 (15)	1.01063 (8)	0.0711 (6)	
H12B	0.7749	−0.2229	1.0233	0.085*	
C13B	0.87748 (13)	−0.17699 (17)	1.00508 (8)	0.0769 (6)	
H13B	0.9026	−0.2329	1.0131	0.092*	
C14B	0.91636 (12)	−0.09989 (17)	0.98765 (8)	0.0736 (6)	
H14B	0.9679	−0.1035	0.9840	0.088*	
C15B	0.87924 (10)	−0.01730 (15)	0.97548 (7)	0.0617 (5)	
H15B	0.9061	0.0351	0.9644	0.074*	
C16B	0.50000 (9)	0.03632 (12)	0.91967 (7)	0.0511 (4)	
H16B	0.4981	−0.0001	0.9494	0.061*	
H16D	0.4733	0.0004	0.8952	0.061*	
C17B	0.46085 (8)	0.13002 (12)	0.92738 (6)	0.0458 (4)	
C18B	0.44536 (10)	0.16354 (14)	0.97336 (7)	0.0588 (5)	
H18B	0.4611	0.1286	1.0000	0.071*	
C19B	0.40708 (11)	0.24777 (15)	0.98023 (8)	0.0698 (6)	
H19B	0.3966	0.2689	1.0113	0.084*	
C20B	0.38446 (11)	0.30048 (14)	0.94116 (8)	0.0667 (6)	
H20B	0.3591	0.3577	0.9456	0.080*	
C21B	0.39945 (10)	0.26832 (14)	0.89542 (8)	0.0636 (5)	
H21B	0.3841	0.3040	0.8689	0.076*	
C22B	0.43716 (10)	0.18334 (13)	0.88838 (7)	0.0546 (4)	
H22B	0.4466	0.1620	0.8572	0.066*	

### Atomic displacement parameters (Å^2^)

**Table:**

	*U*^11^	*U*^22^	*U*^33^	*U*^12^	*U*^13^	*U*^23^
O1A	0.0545 (8)	0.1060 (12)	0.0629 (9)	0.0081 (8)	0.0042 (7)	0.0195 (8)
O2A	0.0782 (10)	0.0927 (11)	0.0600 (8)	0.0061 (8)	−0.0186 (8)	−0.0133 (8)
N1A	0.0497 (8)	0.0526 (8)	0.0470 (8)	−0.0007 (7)	−0.0019 (7)	0.0021 (7)
N3A	0.0485 (8)	0.0514 (9)	0.0592 (9)	−0.0013 (7)	−0.0091 (7)	0.0050 (7)
C2A	0.0508 (10)	0.0543 (11)	0.0564 (11)	0.0043 (8)	−0.0011 (9)	0.0129 (9)
C4A	0.0613 (11)	0.0454 (10)	0.0558 (11)	0.0007 (9)	−0.0080 (10)	−0.0016 (9)
C4AA	0.0562 (10)	0.0357 (8)	0.0509 (10)	0.0001 (8)	−0.0025 (8)	−0.0001 (8)
C5A	0.0755 (13)	0.0490 (11)	0.0544 (11)	−0.0009 (10)	0.0017 (10)	−0.0074 (9)
C6A	0.0621 (12)	0.0599 (12)	0.0699 (13)	−0.0041 (10)	0.0134 (11)	−0.0085 (10)
C7A	0.0521 (11)	0.0594 (12)	0.0765 (14)	−0.0048 (9)	0.0008 (10)	−0.0087 (11)
C8A	0.0519 (10)	0.0581 (11)	0.0589 (11)	−0.0027 (9)	−0.0024 (9)	−0.0011 (10)
C8AA	0.0496 (9)	0.0364 (8)	0.0501 (10)	−0.0015 (7)	0.0003 (8)	0.0025 (8)
C9A	0.0567 (10)	0.0526 (10)	0.0500 (10)	0.0017 (9)	−0.0012 (9)	−0.0030 (9)
C10A	0.0447 (9)	0.0556 (10)	0.0436 (9)	0.0055 (8)	0.0024 (8)	−0.0033 (8)
C11A	0.0571 (11)	0.0617 (12)	0.0561 (11)	0.0052 (9)	−0.0041 (9)	−0.0008 (10)
C12A	0.0811 (15)	0.0588 (12)	0.0766 (14)	0.0096 (11)	0.0074 (12)	0.0109 (11)
C13A	0.0798 (15)	0.0928 (17)	0.0599 (13)	0.0336 (14)	0.0013 (12)	0.0178 (13)
C14A	0.0747 (15)	0.1002 (18)	0.0653 (14)	0.0219 (14)	−0.0215 (12)	−0.0031 (14)
C15A	0.0579 (11)	0.0750 (14)	0.0684 (13)	0.0040 (10)	−0.0115 (10)	−0.0061 (11)
C16A	0.0493 (10)	0.0628 (12)	0.0758 (14)	−0.0107 (9)	−0.0113 (10)	0.0083 (10)
C17A	0.0356 (8)	0.0619 (11)	0.0616 (11)	−0.0065 (8)	−0.0052 (8)	0.0112 (10)
C18A	0.0470 (10)	0.0761 (14)	0.0621 (12)	−0.0012 (10)	0.0051 (9)	0.0156 (11)
C19A	0.0567 (12)	0.0811 (15)	0.0867 (16)	0.0119 (11)	0.0168 (11)	0.0081 (13)
C20A	0.0631 (14)	0.0857 (17)	0.113 (2)	0.0246 (12)	0.0145 (14)	0.0336 (16)
C21A	0.0695 (14)	0.110 (2)	0.0764 (16)	0.0181 (14)	0.0016 (12)	0.0386 (15)
C22A	0.0568 (12)	0.0878 (15)	0.0607 (12)	0.0031 (11)	−0.0043 (10)	0.0106 (12)
O1B	0.0576 (8)	0.0803 (9)	0.0520 (8)	0.0075 (7)	0.0021 (6)	0.0003 (7)
O2B	0.0490 (7)	0.0854 (10)	0.0661 (8)	−0.0013 (7)	−0.0096 (7)	−0.0101 (7)
N1B	0.0412 (7)	0.0463 (8)	0.0525 (8)	0.0014 (6)	−0.0047 (6)	−0.0038 (7)
N3B	0.0388 (7)	0.0430 (8)	0.0545 (9)	0.0020 (6)	−0.0008 (6)	0.0011 (7)
C2B	0.0468 (9)	0.0414 (9)	0.0558 (11)	0.0049 (8)	−0.0025 (8)	0.0012 (8)
C4AB	0.0451 (9)	0.0342 (8)	0.0539 (10)	0.0011 (7)	−0.0002 (8)	−0.0014 (8)
C4B	0.0430 (9)	0.0424 (9)	0.0573 (11)	0.0001 (8)	−0.0052 (8)	−0.0026 (8)
C5B	0.0563 (11)	0.0509 (10)	0.0556 (11)	0.0017 (9)	−0.0021 (9)	−0.0032 (9)
C6B	0.0647 (12)	0.0540 (11)	0.0610 (12)	0.0016 (9)	0.0103 (10)	0.0006 (10)
C7B	0.0469 (10)	0.0493 (10)	0.0766 (14)	−0.0005 (8)	0.0122 (9)	0.0075 (10)
C8AB	0.0410 (9)	0.0329 (8)	0.0567 (10)	−0.0001 (7)	−0.0021 (8)	0.0006 (8)
C8B	0.0426 (9)	0.0503 (10)	0.0670 (12)	−0.0011 (8)	−0.0036 (9)	0.0051 (9)
C9B	0.0515 (10)	0.0515 (10)	0.0605 (11)	0.0012 (8)	−0.0098 (9)	−0.0124 (9)
C10B	0.0493 (10)	0.0514 (10)	0.0486 (10)	0.0019 (8)	−0.0101 (8)	−0.0098 (8)
C11B	0.0569 (11)	0.0610 (12)	0.0584 (11)	0.0023 (9)	−0.0048 (9)	−0.0066 (10)
C12B	0.0818 (15)	0.0609 (13)	0.0704 (13)	0.0033 (11)	−0.0094 (12)	0.0060 (11)
C13B	0.0835 (16)	0.0728 (15)	0.0745 (15)	0.0234 (13)	−0.0169 (13)	0.0020 (12)
C14B	0.0555 (12)	0.0895 (16)	0.0759 (14)	0.0186 (12)	−0.0117 (11)	0.0002 (13)
C15B	0.0499 (11)	0.0696 (13)	0.0654 (12)	0.0003 (10)	−0.0100 (9)	−0.0030 (10)
C16B	0.0393 (9)	0.0478 (10)	0.0662 (12)	−0.0032 (8)	0.0020 (8)	0.0087 (9)
C17B	0.0338 (8)	0.0465 (10)	0.0570 (10)	−0.0023 (7)	0.0011 (8)	0.0081 (8)
C18B	0.0556 (11)	0.0633 (12)	0.0574 (12)	0.0069 (9)	0.0038 (9)	0.0122 (10)
C19B	0.0674 (13)	0.0741 (14)	0.0679 (14)	0.0142 (11)	0.0075 (11)	−0.0061 (12)
C20B	0.0549 (11)	0.0543 (12)	0.0908 (16)	0.0124 (9)	0.0031 (11)	−0.0005 (11)
C21B	0.0558 (11)	0.0586 (12)	0.0764 (14)	0.0083 (9)	−0.0088 (10)	0.0154 (11)
C22B	0.0502 (10)	0.0575 (11)	0.0561 (11)	0.0037 (9)	−0.0032 (9)	0.0078 (9)

### Geometric parameters (Å, °)

**Table:**

O1A—C2A	1.221 (2)	O1B—C2B	1.222 (2)
O2A—C4A	1.220 (2)	O2B—C4B	1.2222 (19)
N1A—C2A	1.368 (2)	N1B—C2B	1.379 (2)
N1A—C8AA	1.397 (2)	N1B—C8AB	1.397 (2)
N1A—C9A	1.474 (2)	N1B—C9B	1.474 (2)
N3A—C4A	1.389 (2)	N3B—C4B	1.387 (2)
N3A—C2A	1.399 (2)	N3B—C2B	1.388 (2)
N3A—C16A	1.478 (2)	N3B—C16B	1.479 (2)
C4A—C4AA	1.461 (2)	C4AB—C8AB	1.389 (2)
C4AA—C8AA	1.393 (2)	C4AB—C5B	1.401 (2)
C4AA—C5A	1.397 (2)	C4AB—C4B	1.455 (2)
C5A—C6A	1.369 (3)	C5B—C6B	1.368 (2)
C5A—H5A	0.9300	C5B—H5B	0.9300
C6A—C7A	1.387 (3)	C6B—C7B	1.377 (3)
C6A—H6A	0.9300	C6B—H6B	0.9300
C7A—C8A	1.374 (2)	C7B—C8B	1.375 (2)
C7A—H7A	0.9300	C7B—H7B	0.9300
C8A—C8AA	1.398 (2)	C8AB—C8B	1.393 (2)
C8A—H8A	0.9300	C8B—H8B	0.9300
C9A—C10A	1.510 (2)	C9B—C10B	1.507 (2)
C9A—H9AA	0.9700	C9B—H9B	0.9700
C9A—H9AB	0.9700	C9B—H9D	0.9700
C10A—C11A	1.379 (2)	C10B—C11B	1.378 (2)
C10A—C15A	1.383 (2)	C10B—C15B	1.392 (2)
C11A—C12A	1.388 (3)	C11B—C12B	1.384 (3)
C11A—H11A	0.9300	C11B—H11B	0.9300
C12A—C13A	1.370 (3)	C12B—C13B	1.376 (3)
C12A—H12A	0.9300	C12B—H12B	0.9300
C13A—C14A	1.359 (3)	C13B—C14B	1.373 (3)
C13A—H13A	0.9300	C13B—H13B	0.9300
C14A—C15A	1.380 (3)	C14B—C15B	1.376 (3)
C14A—H14A	0.9300	C14B—H14B	0.9300
C15A—H15A	0.9300	C15B—H15B	0.9300
C16A—C17A	1.513 (2)	C16B—C17B	1.503 (2)
C16A—H16A	0.9700	C16B—H16B	0.9700
C16A—H16C	0.9700	C16B—H16D	0.9700
C17A—C18A	1.380 (3)	C17B—C22B	1.381 (2)
C17A—C22A	1.384 (3)	C17B—C18B	1.386 (2)
C18A—C19A	1.377 (3)	C18B—C19B	1.378 (2)
C18A—H18A	0.9300	C18B—H18B	0.9300
C19A—C20A	1.374 (3)	C19B—C20B	1.372 (3)
C19A—H19A	0.9300	C19B—H19B	0.9300
C20A—C21A	1.373 (3)	C20B—C21B	1.372 (3)
C20A—H20A	0.9300	C20B—H20B	0.9300
C21A—C22A	1.377 (3)	C21B—C22B	1.382 (2)
C21A—H21A	0.9300	C21B—H21B	0.9300
C22A—H22A	0.9300	C22B—H22B	0.9300
			
C2A—N1A—C8AA	123.03 (15)	C2B—N1B—C8AB	122.66 (14)
C2A—N1A—C9A	116.58 (15)	C2B—N1B—C9B	116.98 (14)
C8AA—N1A—C9A	120.11 (14)	C8AB—N1B—C9B	120.36 (14)
C4A—N3A—C2A	124.81 (15)	C4B—N3B—C2B	125.30 (14)
C4A—N3A—C16A	118.42 (15)	C4B—N3B—C16B	117.80 (14)
C2A—N3A—C16A	116.68 (16)	C2B—N3B—C16B	116.87 (14)
O1A—C2A—N1A	122.15 (17)	O1B—C2B—N1B	122.51 (16)
O1A—C2A—N3A	120.93 (17)	O1B—C2B—N3B	120.76 (16)
N1A—C2A—N3A	116.91 (16)	N1B—C2B—N3B	116.73 (15)
O2A—C4A—N3A	120.45 (18)	C8AB—C4AB—C5B	119.85 (16)
O2A—C4A—C4AA	124.14 (18)	C8AB—C4AB—C4B	120.59 (16)
N3A—C4A—C4AA	115.41 (16)	C5B—C4AB—C4B	119.56 (16)
C8AA—C4AA—C5A	119.31 (16)	O2B—C4B—N3B	120.91 (15)
C8AA—C4AA—C4A	120.56 (16)	O2B—C4B—C4AB	123.68 (17)
C5A—C4AA—C4A	120.13 (16)	N3B—C4B—C4AB	115.40 (15)
C6A—C5A—C4AA	121.04 (18)	C6B—C5B—C4AB	120.52 (17)
C6A—C5A—H5A	119.5	C6B—C5B—H5B	119.7
C4AA—C5A—H5A	119.5	C4AB—C5B—H5B	119.7
C5A—C6A—C7A	119.11 (19)	C5B—C6B—C7B	119.34 (18)
C5A—C6A—H6A	120.4	C5B—C6B—H6B	120.3
C7A—C6A—H6A	120.4	C7B—C6B—H6B	120.3
C8A—C7A—C6A	121.32 (18)	C8B—C7B—C6B	121.30 (18)
C8A—C7A—H7A	119.3	C8B—C7B—H7B	119.4
C6A—C7A—H7A	119.3	C6B—C7B—H7B	119.4
C7A—C8A—C8AA	119.60 (18)	C4AB—C8AB—C8B	118.93 (16)
C7A—C8A—H8A	120.2	C4AB—C8AB—N1B	119.22 (15)
C8AA—C8A—H8A	120.2	C8B—C8AB—N1B	121.84 (15)
C4AA—C8AA—N1A	118.95 (15)	C7B—C8B—C8AB	120.05 (17)
C4AA—C8AA—C8A	119.60 (17)	C7B—C8B—H8B	120.0
N1A—C8AA—C8A	121.45 (16)	C8AB—C8B—H8B	120.0
N1A—C9A—C10A	115.43 (14)	N1B—C9B—C10B	113.29 (14)
N1A—C9A—H9AA	108.4	N1B—C9B—H9B	108.9
C10A—C9A—H9AA	108.4	C10B—C9B—H9B	108.9
N1A—C9A—H9AB	108.4	N1B—C9B—H9D	108.9
C10A—C9A—H9AB	108.4	C10B—C9B—H9D	108.9
H9AA—C9A—H9AB	107.5	H9B—C9B—H9D	107.7
C11A—C10A—C15A	118.66 (18)	C11B—C10B—C15B	118.35 (17)
C11A—C10A—C9A	122.06 (16)	C11B—C10B—C9B	120.66 (16)
C15A—C10A—C9A	119.08 (17)	C15B—C10B—C9B	120.98 (17)
C10A—C11A—C12A	120.43 (19)	C10B—C11B—C12B	120.85 (19)
C10A—C11A—H11A	119.8	C10B—C11B—H11B	119.6
C12A—C11A—H11A	119.8	C12B—C11B—H11B	119.6
C13A—C12A—C11A	119.9 (2)	C13B—C12B—C11B	120.0 (2)
C13A—C12A—H12A	120.1	C13B—C12B—H12B	120.0
C11A—C12A—H12A	120.1	C11B—C12B—H12B	120.0
C14A—C13A—C12A	120.2 (2)	C14B—C13B—C12B	119.8 (2)
C14A—C13A—H13A	119.9	C14B—C13B—H13B	120.1
C12A—C13A—H13A	119.9	C12B—C13B—H13B	120.1
C13A—C14A—C15A	120.3 (2)	C13B—C14B—C15B	120.2 (2)
C13A—C14A—H14A	119.8	C13B—C14B—H14B	119.9
C15A—C14A—H14A	119.8	C15B—C14B—H14B	119.9
C14A—C15A—C10A	120.5 (2)	C14B—C15B—C10B	120.8 (2)
C14A—C15A—H15A	119.7	C14B—C15B—H15B	119.6
C10A—C15A—H15A	119.7	C10B—C15B—H15B	119.6
N3A—C16A—C17A	111.85 (14)	N3B—C16B—C17B	113.76 (13)
N3A—C16A—H16A	109.2	N3B—C16B—H16B	108.8
C17A—C16A—H16A	109.2	C17B—C16B—H16B	108.8
N3A—C16A—H16C	109.2	N3B—C16B—H16D	108.8
C17A—C16A—H16C	109.2	C17B—C16B—H16D	108.8
H16A—C16A—H16C	107.9	H16B—C16B—H16D	107.7
C18A—C17A—C22A	118.84 (19)	C22B—C17B—C18B	118.39 (16)
C18A—C17A—C16A	121.02 (17)	C22B—C17B—C16B	120.26 (16)
C22A—C17A—C16A	120.13 (19)	C18B—C17B—C16B	121.31 (16)
C19A—C18A—C17A	120.79 (19)	C19B—C18B—C17B	121.10 (18)
C19A—C18A—H18A	119.6	C19B—C18B—H18B	119.5
C17A—C18A—H18A	119.6	C17B—C18B—H18B	119.5
C20A—C19A—C18A	119.7 (2)	C20B—C19B—C18B	119.9 (2)
C20A—C19A—H19A	120.1	C20B—C19B—H19B	120.0
C18A—C19A—H19A	120.1	C18B—C19B—H19B	120.0
C21A—C20A—C19A	120.2 (2)	C19B—C20B—C21B	119.68 (18)
C21A—C20A—H20A	119.9	C19B—C20B—H20B	120.2
C19A—C20A—H20A	119.9	C21B—C20B—H20B	120.2
C20A—C21A—C22A	120.1 (2)	C20B—C21B—C22B	120.58 (19)
C20A—C21A—H21A	120.0	C20B—C21B—H21B	119.7
C22A—C21A—H21A	120.0	C22B—C21B—H21B	119.7
C21A—C22A—C17A	120.4 (2)	C17B—C22B—C21B	120.34 (18)
C21A—C22A—H22A	119.8	C17B—C22B—H22B	119.8
C17A—C22A—H22A	119.8	C21B—C22B—H22B	119.8
			
C8AA—N1A—C2A—O1A	−175.32 (16)	C8AB—N1B—C2B—O1B	177.61 (15)
C9A—N1A—C2A—O1A	10.7 (3)	C9B—N1B—C2B—O1B	−1.9 (2)
C8AA—N1A—C2A—N3A	5.7 (3)	C8AB—N1B—C2B—N3B	−1.8 (2)
C9A—N1A—C2A—N3A	−168.28 (14)	C9B—N1B—C2B—N3B	178.73 (13)
C4A—N3A—C2A—O1A	−179.83 (17)	C4B—N3B—C2B—O1B	179.24 (16)
C16A—N3A—C2A—O1A	−3.4 (3)	C16B—N3B—C2B—O1B	−2.5 (2)
C4A—N3A—C2A—N1A	−0.8 (3)	C4B—N3B—C2B—N1B	−1.4 (2)
C16A—N3A—C2A—N1A	175.60 (15)	C16B—N3B—C2B—N1B	176.87 (13)
C2A—N3A—C4A—O2A	177.49 (17)	C2B—N3B—C4B—O2B	−178.43 (16)
C16A—N3A—C4A—O2A	1.1 (3)	C16B—N3B—C4B—O2B	3.4 (2)
C2A—N3A—C4A—C4AA	−3.4 (3)	C2B—N3B—C4B—C4AB	2.9 (2)
C16A—N3A—C4A—C4AA	−179.81 (14)	C16B—N3B—C4B—C4AB	−175.28 (13)
O2A—C4A—C4AA—C8AA	−177.75 (17)	C8AB—C4AB—C4B—O2B	179.88 (16)
N3A—C4A—C4AA—C8AA	3.2 (2)	C5B—C4AB—C4B—O2B	−0.7 (3)
O2A—C4A—C4AA—C5A	1.8 (3)	C8AB—C4AB—C4B—N3B	−1.5 (2)
N3A—C4A—C4AA—C5A	−177.27 (15)	C5B—C4AB—C4B—N3B	177.94 (15)
C8AA—C4AA—C5A—C6A	−0.2 (3)	C8AB—C4AB—C5B—C6B	1.3 (3)
C4A—C4AA—C5A—C6A	−179.71 (17)	C4B—C4AB—C5B—C6B	−178.20 (16)
C4AA—C5A—C6A—C7A	−1.2 (3)	C4AB—C5B—C6B—C7B	−0.9 (3)
C5A—C6A—C7A—C8A	1.1 (3)	C5B—C6B—C7B—C8B	−0.2 (3)
C6A—C7A—C8A—C8AA	0.3 (3)	C5B—C4AB—C8AB—C8B	−0.5 (2)
C5A—C4AA—C8AA—N1A	−178.40 (15)	C4B—C4AB—C8AB—C8B	178.98 (15)
C4A—C4AA—C8AA—N1A	1.1 (2)	C5B—C4AB—C8AB—N1B	179.26 (15)
C5A—C4AA—C8AA—C8A	1.6 (3)	C4B—C4AB—C8AB—N1B	−1.3 (2)
C4A—C4AA—C8AA—C8A	−178.84 (16)	C2B—N1B—C8AB—C4AB	3.1 (2)
C2A—N1A—C8AA—C4AA	−5.9 (2)	C9B—N1B—C8AB—C4AB	−177.48 (14)
C9A—N1A—C8AA—C4AA	167.91 (15)	C2B—N1B—C8AB—C8B	−177.20 (15)
C2A—N1A—C8AA—C8A	174.10 (16)	C9B—N1B—C8AB—C8B	2.3 (2)
C9A—N1A—C8AA—C8A	−12.1 (2)	C6B—C7B—C8B—C8AB	0.9 (3)
C7A—C8A—C8AA—C4AA	−1.7 (3)	C4AB—C8AB—C8B—C7B	−0.6 (2)
C7A—C8A—C8AA—N1A	178.33 (16)	N1B—C8AB—C8B—C7B	179.66 (15)
C2A—N1A—C9A—C10A	−99.51 (18)	C2B—N1B—C9B—C10B	102.55 (18)
C8AA—N1A—C9A—C10A	86.33 (19)	C8AB—N1B—C9B—C10B	−76.95 (19)
N1A—C9A—C10A—C11A	38.0 (2)	N1B—C9B—C10B—C11B	−60.1 (2)
N1A—C9A—C10A—C15A	−147.14 (17)	N1B—C9B—C10B—C15B	120.61 (18)
C15A—C10A—C11A—C12A	0.1 (3)	C15B—C10B—C11B—C12B	−0.2 (3)
C9A—C10A—C11A—C12A	174.99 (16)	C9B—C10B—C11B—C12B	−179.58 (17)
C10A—C11A—C12A—C13A	0.0 (3)	C10B—C11B—C12B—C13B	−1.5 (3)
C11A—C12A—C13A—C14A	−0.5 (3)	C11B—C12B—C13B—C14B	1.8 (3)
C12A—C13A—C14A—C15A	0.9 (3)	C12B—C13B—C14B—C15B	−0.4 (3)
C13A—C14A—C15A—C10A	−0.8 (3)	C13B—C14B—C15B—C10B	−1.4 (3)
C11A—C10A—C15A—C14A	0.3 (3)	C11B—C10B—C15B—C14B	1.7 (3)
C9A—C10A—C15A—C14A	−174.73 (18)	C9B—C10B—C15B—C14B	−178.98 (17)
C4A—N3A—C16A—C17A	88.4 (2)	C4B—N3B—C16B—C17B	−97.31 (18)
C2A—N3A—C16A—C17A	−88.3 (2)	C2B—N3B—C16B—C17B	84.33 (18)
N3A—C16A—C17A—C18A	81.3 (2)	N3B—C16B—C17B—C22B	76.8 (2)
N3A—C16A—C17A—C22A	−97.6 (2)	N3B—C16B—C17B—C18B	−105.44 (19)
C22A—C17A—C18A—C19A	2.4 (3)	C22B—C17B—C18B—C19B	0.2 (3)
C16A—C17A—C18A—C19A	−176.51 (17)	C16B—C17B—C18B—C19B	−177.63 (17)
C17A—C18A—C19A—C20A	−2.0 (3)	C17B—C18B—C19B—C20B	−0.8 (3)
C18A—C19A—C20A—C21A	0.1 (3)	C18B—C19B—C20B—C21B	0.7 (3)
C19A—C20A—C21A—C22A	1.4 (4)	C19B—C20B—C21B—C22B	0.0 (3)
C20A—C21A—C22A—C17A	−0.9 (3)	C18B—C17B—C22B—C21B	0.5 (3)
C18A—C17A—C22A—C21A	−1.0 (3)	C16B—C17B—C22B—C21B	178.34 (16)
C16A—C17A—C22A—C21A	177.99 (18)	C20B—C21B—C22B—C17B	−0.6 (3)

### Hydrogen-bond geometry (Å, °)

**Table:**

Cg4 and Cg8 are the centroids of the C17A–C22A and C10B–C15B rings, respectively.

**Table:**

*D*—H···*A*	*D*—H	H···*A*	*D*···*A*	*D*—H···*A*
C19A—H19A···O2b^i^	0.93	2.70	3.419 (3)	134
C6B—H6B···C11a^ii^	0.93	2.89	3.604 (3)	134
C21B—H21B···C11a^iii^	0.93	2.80	3.600 (3)	145
C19A—H19A···O2b^i^	0.93	2.70	3.419 (3)	134
C7B—H7B···Cg4	0.93	2.78	3.586 (2)	146
C5A—H5A···Cg8^ii^	0.93	2.90	3.641 (2)	138

Symmetry codes: (i) *x*+1/2, *y*, −*z*+3/2; (ii) −*x*+3/2, *y*−1/2, *z*; (iii) *x*−1/2, *y*, −*z*+3/2.
